# Carbapenemase type-dependent pharmacodynamic activity of minocycline–biapenem combination against carbapenemase-producing *Klebsiella pneumoniae*

**DOI:** 10.1093/jacamr/dlag168

**Published:** 2026-08-06

**Authors:** Takashi Okanda, Niinyo Nakajima, Tetsuo Yamaguchi, Takuro Koshikawa, Tadatomo Oyanagi, Tomonori Takano, Hiroyuki Kunishima, Mitsuo Kaku, Hiromu Takemura

**Affiliations:** Department of Microbiology, St. Marianna University School of Medicine, 2-16-1 Sugao, Miyamae-ku, Kawasaki, Kanagawa 216-8511, Japan; Department of Microbiology, St. Marianna University School of Medicine, 2-16-1 Sugao, Miyamae-ku, Kawasaki, Kanagawa 216-8511, Japan; Department of Microbiology, St. Marianna University School of Medicine, 2-16-1 Sugao, Miyamae-ku, Kawasaki, Kanagawa 216-8511, Japan; Department of Microbiology, St. Marianna University School of Medicine, 2-16-1 Sugao, Miyamae-ku, Kawasaki, Kanagawa 216-8511, Japan; Department of Clinical Laboratory Technology, St. Marianna University Hospital, Kawasaki, Kanagawa, Japan; Department of Infectious Diseases, St. Marianna University School of Medicine, Kawasaki, Kanagawa, Japan; Department of Infectious Diseases, St. Marianna University School of Medicine, Kawasaki, Kanagawa, Japan; Department of Infectious Diseases, St. Marianna University School of Medicine, Kawasaki, Kanagawa, Japan; Department of Microbiology, St. Marianna University School of Medicine, 2-16-1 Sugao, Miyamae-ku, Kawasaki, Kanagawa 216-8511, Japan

## Abstract

**Objectives:**

The activity of antimicrobial combinations against carbapenemase-producing *Klebsiella pneumoniae* (CPKP) is likely to depend on the underlying resistance mechanism; however, this has not been systematically evaluated. We assessed the *in vitro* activity of minocycline in combination with biapenem across major carbapenemase types.

**Methods:**

A total of 79 clinical CPKP isolates from Japan (*n* = 30) and Bangladesh (*n* = 49), including New Delhi metallo-β-lactamase (NDM) (*n* = 40), imipenemase (IMP) (*n* = 10), *K. pneumoniae* carbapenemase (KPC) (*n* = 10), oxacillinase-48-like (OXA-48-like) (*n* = 16) and NDM+OXA-48-like (*n* = 3), were analysed. MICs were determined by broth microdilution in accordance with CLSI guidelines. Combination activity was evaluated using checkerboard assays to calculate fractional inhibitory concentration indices (FICIs), and cumulative growth inhibition was analysed across concentration matrices.

**Results:**

Single-agent MIC90 values of minocycline were 32, 16, 8, 32 and 32 mg/L for NDM, IMP, KPC, OXA-48-like and NDM+OXA-48-like isolates, respectively; corresponding biapenem MIC90 values were 32, 2, 256, 8 and 256 mg/L. Synergistic interactions (FICI ≤0.5) with the minocycline–biapenem combination were observed against 98% of NDM, 80% of IMP, 60% of KPC, 38% of OXA-48-like and 100% of NDM+OXA-48-like isolates. At minocycline 4 mg/L plus biapenem 1 mg/L, inhibition rates were 98%, 100%, 80% and 63% for NDM, IMP, KPC and OXA-48-like isolates, respectively.

**Conclusions:**

The activity of the minocycline–biapenem combination differs substantially according to carbapenemase type. These findings indicate that combination efficacy is not predictable from single-agent susceptibility alone and highlight the importance of pharmacodynamic evaluation when optimizing treatment strategies for CPKP.

## Introduction

Carbapenemase-producing *Klebsiella pneumoniae* (CPKP) has emerged as a major global public health threat and is currently classified among the highest priority antibiotic-resistant pathogens by the World Health Organization.^1^ Recent epidemiological studies have demonstrated a continued increase in carbapenem-resistant Enterobacterales (CRE) worldwide, with high mortality rates and limited therapeutic options, particularly in infections caused by *K. pneumoniae*.^2–5^ In addition, the epidemiology of CRE varies substantially across geographic regions and healthcare settings, reflecting differences in dominant resistance mechanisms and local transmission dynamics.^3^ In Japan, imipenemase (IMP)-type carbapenemases have historically predominated, whereas other carbapenemase types such as New Delhi metallo-β-lactamase (NDM) are also increasingly detected in recent studies.^4^

Carbapenemases include metallo-β-lactamases (MBLs), such as NDM and IMP, and serine carbapenemases, such as *K. pneumoniae* carbapenemase (KPC) and oxacillinase-48-like (OXA-48-like) enzymes, which differ in their hydrolytic mechanisms and resistance profiles.^6^ These differences may influence not only the activity of individual antimicrobial agents but also the effectiveness of combination therapies.

Minocycline, a tetracycline derivative that inhibits bacterial protein synthesis, has been reconsidered as a potential therapeutic option against multidrug-resistant Gram-negative bacteria. Biapenem is a carbapenem used clinically in Japan and other Asian countries, and pharmacokinetic/pharmacodynamic studies have provided a basis for evaluating clinically relevant exposure thresholds.^7–9^ Although biapenem, like other carbapenems, is expected to have limited standalone activity against carbapenemase-producing organisms, residual β-lactam activity may still contribute to combination effects when paired with an agent with a complementary mechanism of action.

In parallel, combination strategies using agents with limited standalone activity have gained attention as a means of restoring antibacterial efficacy against resistant pathogens.^10,11^ Although minocycline and biapenem are not recommended as standard therapy for CRE infections in current IDSA and ESCMID guidelines, combinations of established agents may remain relevant as potential salvage or regionally accessible options, particularly where novel agents are unavailable, inactive or unsuitable.^12,13^ We previously demonstrated the *in vitro* synergistic activity of minocycline in combination with biapenem against cefiderocol-resistant NDM-producing Enterobacterales.^14^ However, that study focused on a specific resistance phenotype and did not address whether the observed combination effect could be generalized across different carbapenemase types or whether pharmacodynamic interactions differ depending on the underlying resistance mechanism.

The evaluation of antimicrobial combinations has traditionally relied on fractional inhibitory concentration indices (FICIs) derived from checkerboard assays.^15^ Although useful for categorizing interactions, FICI values represent only a single point estimate and may not fully capture antibacterial activity across a range of concentration combinations,^11,15^ particularly when concentration-dependent effects are present.

In this study, we evaluated the *in vitro* activity of minocycline in combination with biapenem against a collection of CPKP clinical isolates representing major carbapenemase types. By integrating conventional synergy testing with cumulative growth inhibition analysis, we aimed to determine whether the activity of this combination differs according to carbapenemase type.

## Materials and methods

### Bacterial isolates

A total of 79 non-duplicate CPKP isolates were included in this study (Table 1). These isolates were recovered from clinical specimens in Japan (*n* = 30) and Bangladesh (*n* = 49). The collection comprised isolates producing NDM (*n* = 40), IMP (*n* = 10), KPC (*n* = 10) and OXA-48-like (*n* = 16) and isolates co-producing NDM and OXA-48-like enzymes (*n* = 3).

**Table 1.** Distribution of carbapenemase types among CPKP isolates (*n* = 79)

Carbapenemase type	No. of isolates, *n* (%)	Japan, *n*	Bangladesh, *n*
NDM	40 (51)	6	34
IMP	10 (13)	10	0
KPC	10 (13)	10	0
OXA-48-like	16 (20)	4	12
NDM+OXA-48-like	3 (4)	0	3
Total	79 (100)	30	49

The number of isolates from Japan and Bangladesh is shown for each carbapenemase group.

A subset of the Bangladeshi isolates has been previously characterized with respect to molecular epidemiology.^16^ However, the present study focuses on phenotypic susceptibility and combination activity, and all isolates were re-evaluated under standardized conditions.

### Antimicrobial susceptibility testing

Minimum inhibitory concentrations (MICs) of minocycline and biapenem were determined by the broth microdilution method in accordance with Clinical and Laboratory Standards Institute (CLSI) guidelines.^17^  *Escherichia coli* ATCC 25922 was used for quality control. Minocycline susceptibility was interpreted according to CLSI criteria.^18^ As no official breakpoint exists for biapenem, a tentative susceptibility threshold of ≤1 mg/L was used, based on pharmacokinetic/pharmacodynamic modelling and clinical data from Asia.^7–9^

### Checkerboard assay and FICI determination

Checkerboard assays were performed on CPKP isolates using 2-fold serial dilutions of minocycline (0.03–64 mg/L) and biapenem (0.03–64 mg/L). FICIs were calculated as FICI = (MIC_A_ in combination/MIC_A_ alone) + (MIC_B_ in combination/MIC_B_ alone). For each isolate, the concentration pair at which the minimum FICI was observed was identified from the checkerboard assays.

### Cumulative growth inhibition analysis

To further evaluate the concentration-dependent activity of the combination, cumulative growth inhibition rates were calculated for each concentration pair of minocycline and biapenem. For each concentration pair, the proportion of isolates exhibiting growth inhibition was calculated. These data were visualized as heatmaps for each carbapenemase group.

### Data analysis

All measurements were performed in triplicate, and median values were used for analysis. Synergistic interactions were defined as FICI ≤0.5, no interaction as >0.5–4.0 and antagonism as >4.0.^15^

## Results

### *In vitro* activity of minocycline and biapenem

**Figure 1.**
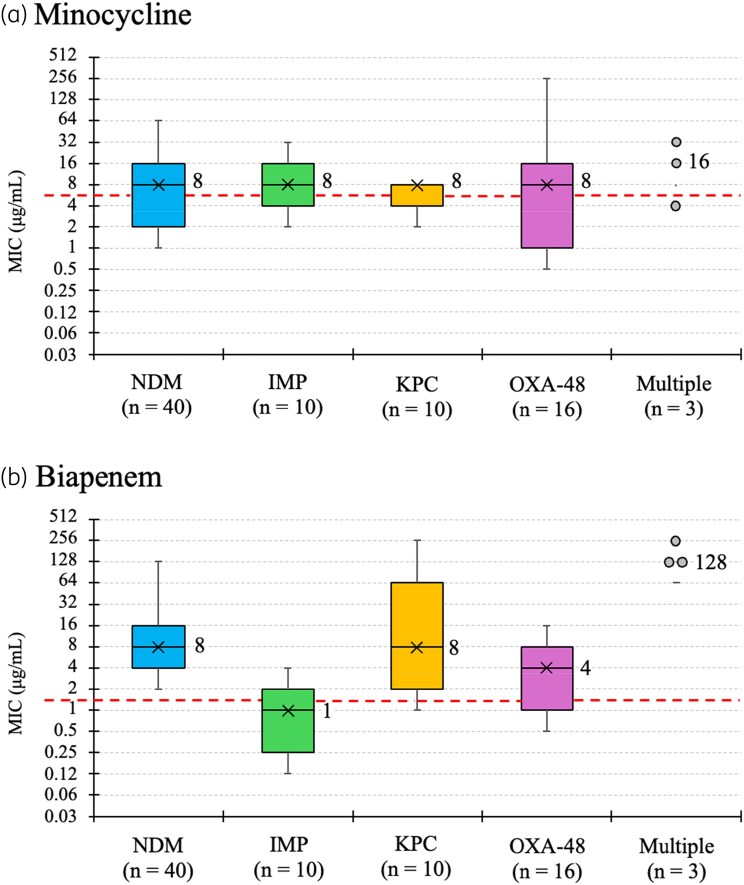
Distribution of MICs of minocycline (a) and biapenem (b) against carbapenemase-producing *Klebsiella pneumoniae* isolates according to carbapenemase type. Each box represents the interquartile range, with the median shown as a horizontal line. Whiskers indicate the range of observed MIC values, and individual outliers are shown as dots. MIC values are presented on a log2 scale. The horizontal dashed line indicates a concentration of 4 mg/L for minocycline (a) and 1 mg/L for biapenem (b). Crosses indicate mean MIC values.

The distributions of MICs for minocycline and biapenem according to carbapenemase type are shown in Figure 1, and summary data are provided in Table 2. For minocycline, MIC distributions were broadly similar across carbapenemase groups, with MIC_50_ values of 8 mg/L for NDM, IMP and OXA-48-like producers and 4 mg/L for KPC producers (Figure 1a). Susceptibility rates ranged from 30% to 50%, with no marked differences among groups (Table 2). For biapenem, MIC distributions varied across carbapenemase types (Figure 1b). IMP-producing isolates showed lower MIC values (MIC_50_, 1 mg/L) compared with other groups, whereas higher MICs were observed in NDM- and KPC-producing isolates (MIC_50_, 8 mg/L and 4 mg/L, respectively). The proportion of isolates with MICs ≤1 mg/L ranged from 8% in NDM producers to 60% in IMP producers (Table 2).

**Table 2.** *In vitro* activity of minocycline and biapenem against carbapenemase-producing *Klebsiella pneumoniae* isolates according to carbapenemase type

	Minocycline				Biapenem			
Carbapenemase type (*n*)	MIC range	MIC_50_	MIC_90_	MIC ≤4 mg/L, *n* (%)	MIC range	MIC_50_	MIC_90_	MIC ≤1 mg/L, *n* (%)
NDM (40)	0.06–64	8	32	17 (43%)	0.25–128	8	32	3 (8%)
IMP (10)	2–32	8	16	3 (30%)	0.12–4	1	2	6 (60%)
KPC (10)	2–8	4	8	5 (50%)	1–256	4	256	1 (10%)
OXA-48-like (16)	1–256	8	32	6 (38%)	0.12–16	4	8	6 (38%)
NDM+OXA-48-like (3)	4–32	16	32	1 (33%)	128–256	128	256	0 (0%)
Total (79)	0.06–256	8	32	32 (41%)	0.12–256	4	32	16 (20%)

Minimum inhibitory concentrations (MICs) were determined by broth microdilution. MIC_50_ and MIC_90_ represent the MICs at which 50% and 90% of isolates were inhibited, respectively. For minocycline, the proportion of isolates with MICs ≤4 mg/L is shown based on CLSI interpretive criteria. Because established clinical breakpoints for biapenem are limited, the proportion of isolates with MICs ≤1 mg/L is presented as a pharmacodynamically relevant threshold.

### Distribution of FICI values

**Figure 2.**
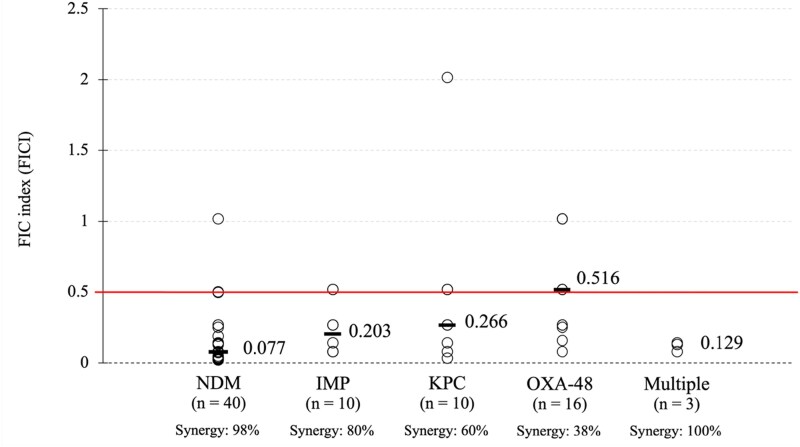
Scatter plot of FICIs for the combination of minocycline and biapenem against carbapenemase-producing *Klebsiella pneumoniae* isolates according to carbapenemase type. Each dot represents an individual isolate. The horizontal line indicates the threshold for synergy (FICI ≤0.5). Median FICI values are shown for each group, and synergy rates are indicated below each category.

The distribution of FICIs for the combination of minocycline and biapenem is shown in Figure 2. Median FICI values differed across carbapenemase types, with lower values observed in NDM- and IMP-producing isolates than in KPC- and OXA-48-like-producing isolates. The median FICI values were 0.077 for NDM, 0.203 for IMP, 0.266 for KPC, 0.516 for OXA-48-like and 0.129 for NDM+OXA-48-like isolates. Synergistic interactions (FICI ≤0.5) were observed against 100% of NDM+OXA-48-like isolates (3/3), 98% of NDM isolates (39/40), 80% of IMP isolates (8/10), 60% of KPC isolates (6/10) and 38% of OXA-48-like isolates (6/16). Because the NDM+OXA-48-like group included only three isolates, this group was interpreted descriptively.

### Cumulative growth inhibition profiles

**Figure 3.**
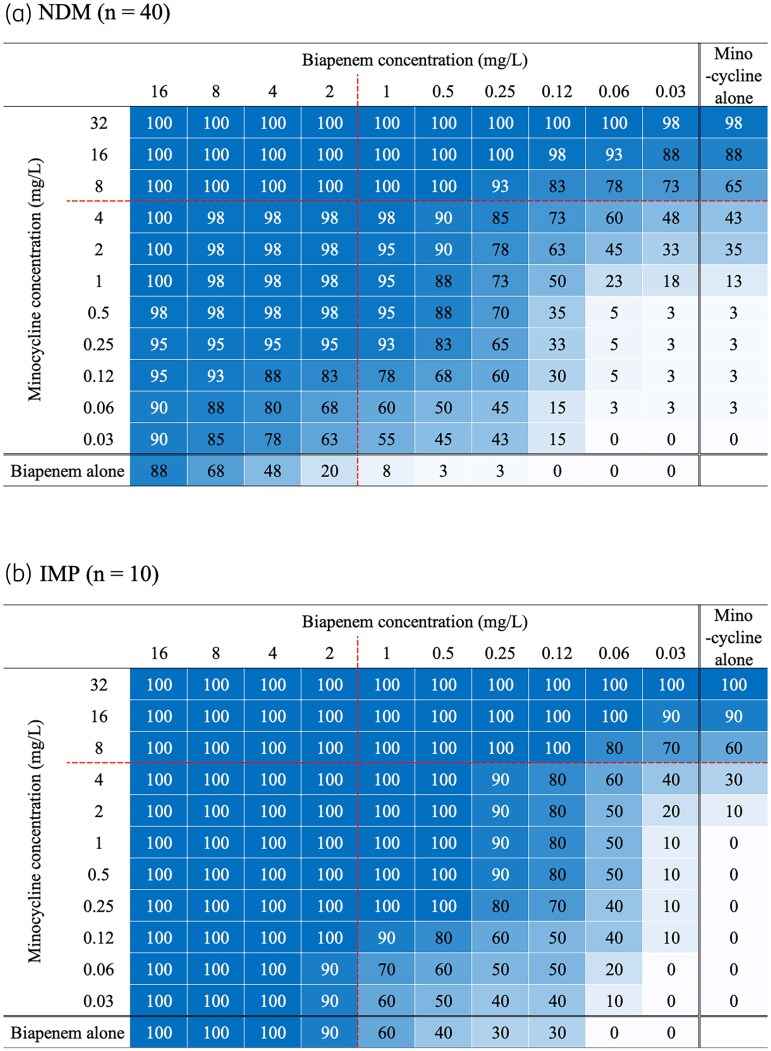

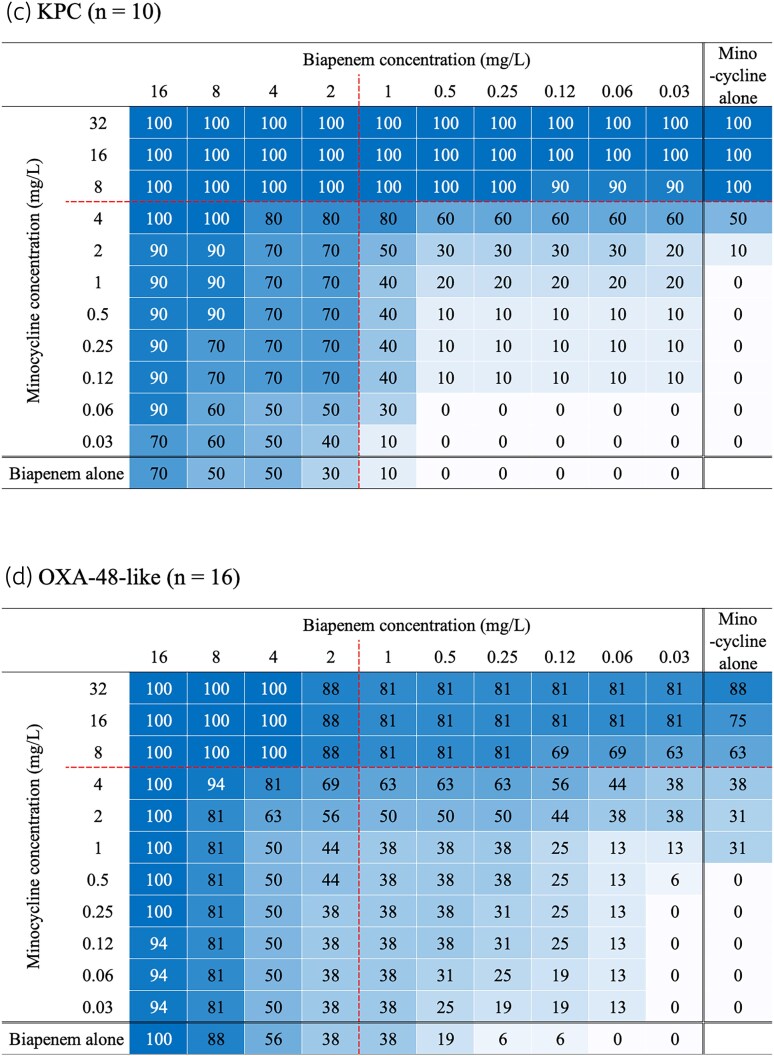
Heatmaps of cumulative growth inhibition rates for the combination of minocycline and biapenem against carbapenemase-producing *Klebsiella pneumoniae* isolates according to carbapenemase type. Each cell represents the percentage of isolates showing no visible growth at the indicated concentrations of minocycline (*y*-axis) and biapenem (*x*-axis). The dashed lines indicate clinically relevant concentrations (minocycline 4 mg/L and biapenem 1 mg/L). The rightmost column and bottom row show inhibition rates for minocycline and biapenem alone, respectively.

Cumulative growth inhibition rates for combinations of minocycline and biapenem are shown in Figure 3. For NDM- and IMP-producing isolates, high inhibition rates were observed across a wide range of concentration combinations, including both higher and lower concentrations of minocycline and biapenem. In contrast, for KPC- and OXA-48-like-producing isolates, high inhibition rates were limited to specific concentration combinations and decreased progressively as drug concentrations were reduced. At clinically relevant concentrations (minocycline 4 mg/L and biapenem 1 mg/L), cumulative inhibition rates were 98% for NDM, 100% for IMP, 80% for KPC and 63% for OXA-48-like isolates.

## Discussion

The present study demonstrated that the *in vitro* activity of minocycline in combination with biapenem against CPKP differed substantially according to carbapenemase type. Although single-agent MIC distributions showed some variability, these differences alone did not fully account for the variation observed in combination activity. Lower FICI values and more frequent synergistic interactions were observed for the minocycline–biapenem combination against NDM- and IMP-producing isolates than against KPC- and OXA-48-like-producing isolates, consistent with previous observations that the effectiveness of combination therapy is likely to depend on the underlying resistance mechanism rather than single-agent susceptibility alone.^10,11^

The cumulative growth inhibition analysis further highlighted these differences. Unlike FICI values, which reflect interactions at a single concentration point, cumulative inhibition profiles represent the proportion of isolates inhibited across a range of concentration combinations.^11,15^ In the present study, cumulative inhibition profiles for NDM- and IMP-producing isolates showed broader areas of high inhibition across the concentration matrix, whereas those for KPC- and OXA-48-like-producing isolates showed more restricted inhibition patterns. Notably, at clinically relevant concentrations (minocycline 4 mg/L and biapenem 1 mg/L), cumulative inhibition rates exceeded 90% for NDM- and IMP-producing isolates, while lower inhibition rates were observed in other carbapenemase groups. These findings indicate that conventional FICI-based assessments may underestimate the extent of combination activity when concentration-dependent effects are present. Importantly, these results suggest that reliance on single-agent MICs or FICI alone may be insufficient to guide combination therapy against CPKP.

These findings should be interpreted in the context of the long-standing distinction between bacteriostatic and bactericidal agents. Although minocycline is generally classified as a bacteriostatic antibiotic, clinical evidence has demonstrated that bacteriostatic agents are not intrinsically inferior to bactericidal agents in terms of clinical efficacy. A systematic review of randomized controlled trials found no consistent difference in outcomes between bacteriostatic and bactericidal antibiotics, highlighting that factors such as pharmacokinetics, pharmacodynamics and drug exposure are more important determinants of therapeutic efficacy.^19^

In this context, the concentration-dependent activity observed in the present study, particularly the broad inhibition patterns observed for NDM- and IMP-producing isolates, suggests that the effectiveness of the minocycline–biapenem combination is better explained by pharmacodynamic interactions than by the intrinsic classification of the agents. The observed differences between carbapenemase types likely reflect differences in residual β-lactam activity in the presence of carbapenemases, which could influence the extent to which protein synthesis inhibition enhances antibacterial effects across concentration ranges. In this regard, biapenem should not be interpreted as a carbapenem expected to retain reliable standalone activity against CPKP, but rather as a β-lactam component whose residual activity may be amplified in the presence of minocycline. This interpretation is consistent with the low proportion of isolates with biapenem MICs ≤1 mg/L in several carbapenemase groups and with the marked differences observed only under combination exposure. Such differences are consistent with the distinct biochemical properties of MBLs and serine carbapenemases, which may influence residual β-lactam activity and, consequently, combination effects.^6^

From a clinical perspective, these findings are particularly relevant in regions such as Japan, where IMP-type carbapenemases have historically predominated, while NDM has also been increasingly recognized in recent years. Although minocycline and biapenem are not currently recommended as standard treatment options for CRE infections in IDSA and ESCMID guidelines, the present findings may be useful for identifying resistance-mechanism-informed combination strategies using regionally available agents.^12,13^ The high cumulative inhibition observed against both IMP- and NDM-producing isolates suggests that the minocycline–biapenem combination may warrant further pharmacodynamic and translational evaluation for infections caused by MBL-producing CPKP. In contrast, the more limited inhibition observed against KPC- and OXA-48-like-producing isolates indicates that the expected benefit of this combination may vary depending on the underlying resistance mechanism.

This study has several limitations. First, the number of isolates in some carbapenemase groups was limited. Second, isolates co-producing NDM and OXA-48-like enzymes were few and were therefore interpreted descriptively; findings in this subgroup should not be over-emphasized. Third, the present analysis was based on *in vitro* checkerboard-derived data, and no time–kill or *in vivo* validation was performed. Finally, epidemiological differences between isolates from Japan and Bangladesh may have influenced the observed patterns.

In conclusion, the activity of the minocycline–biapenem combination differed according to carbapenemase type, with more consistent and concentration-dependent inhibition observed in NDM- and IMP-producing isolates. These findings suggest that the effectiveness of this combination is determined not only by baseline susceptibility but also by pharmacodynamic interactions across concentration ranges, highlighting the importance of mechanism-informed combination strategies.
